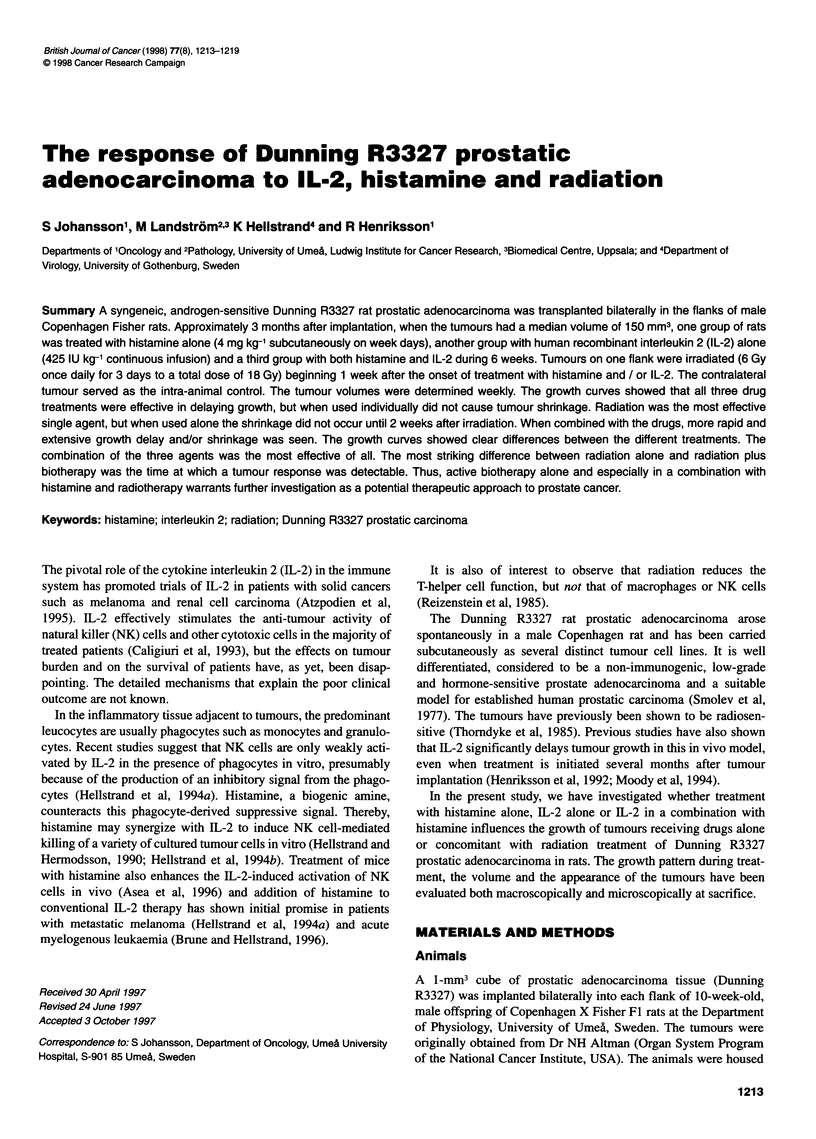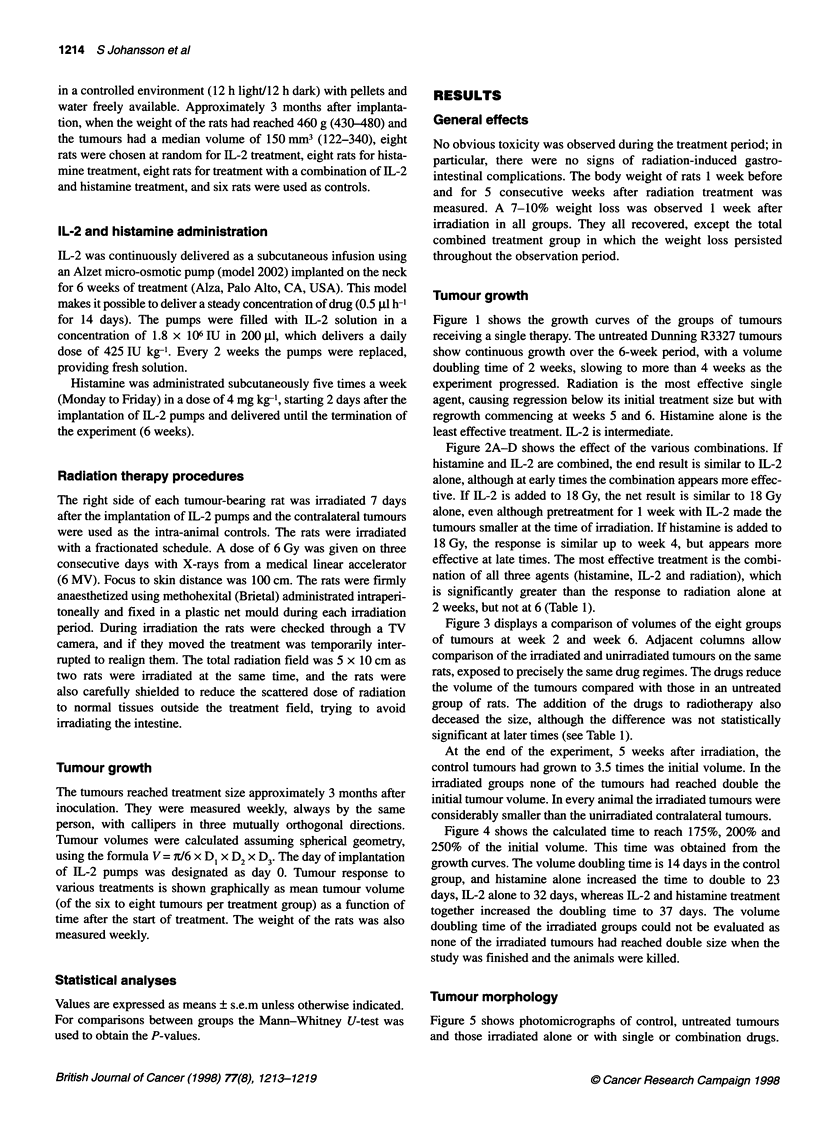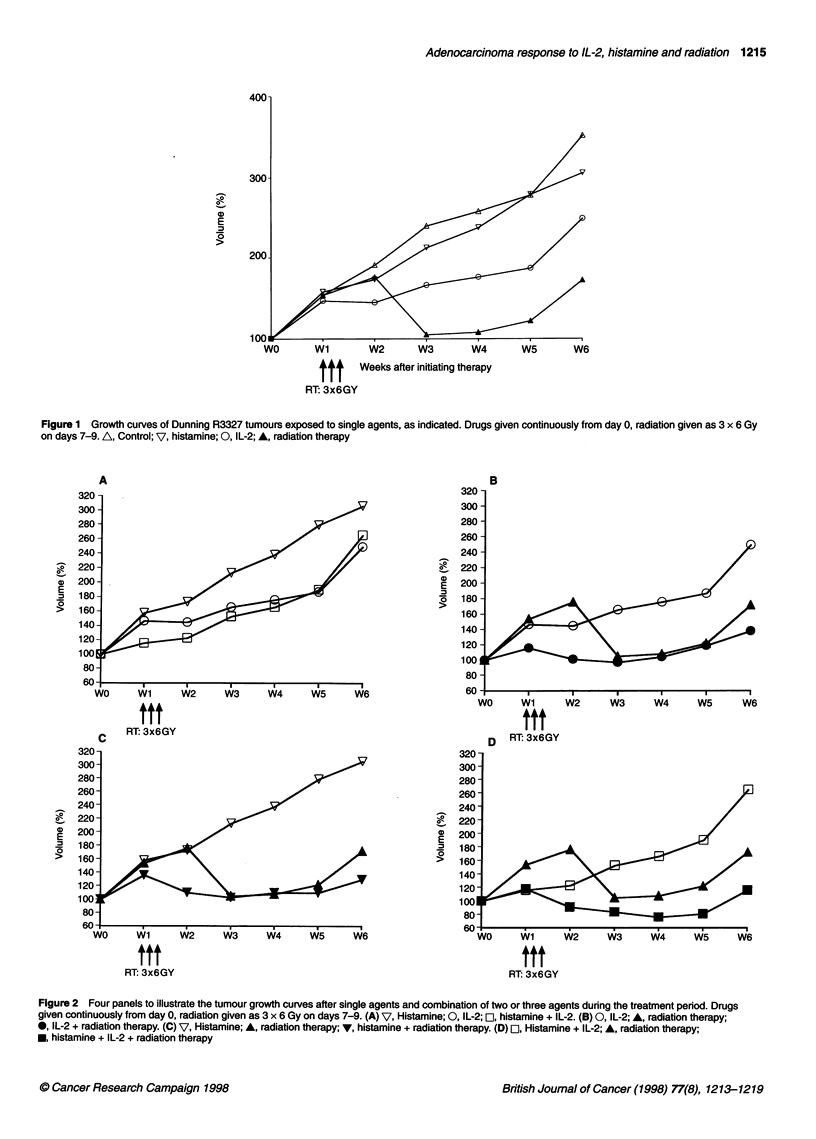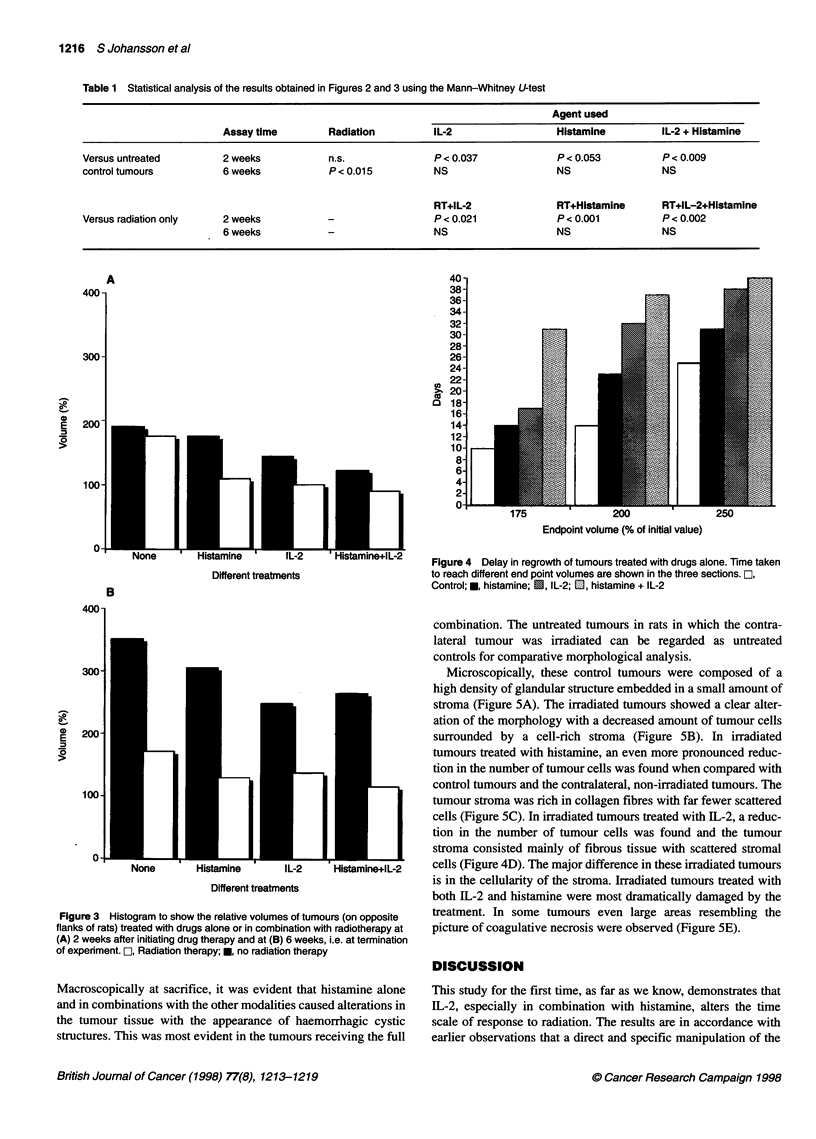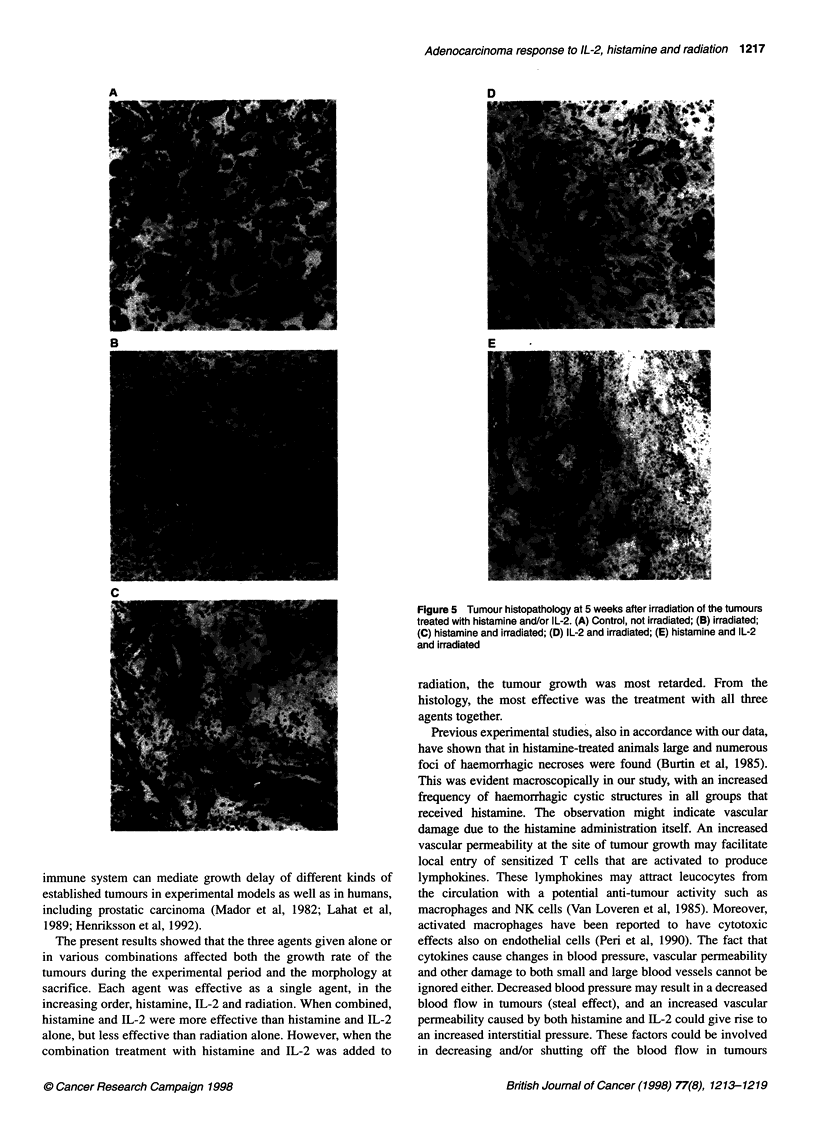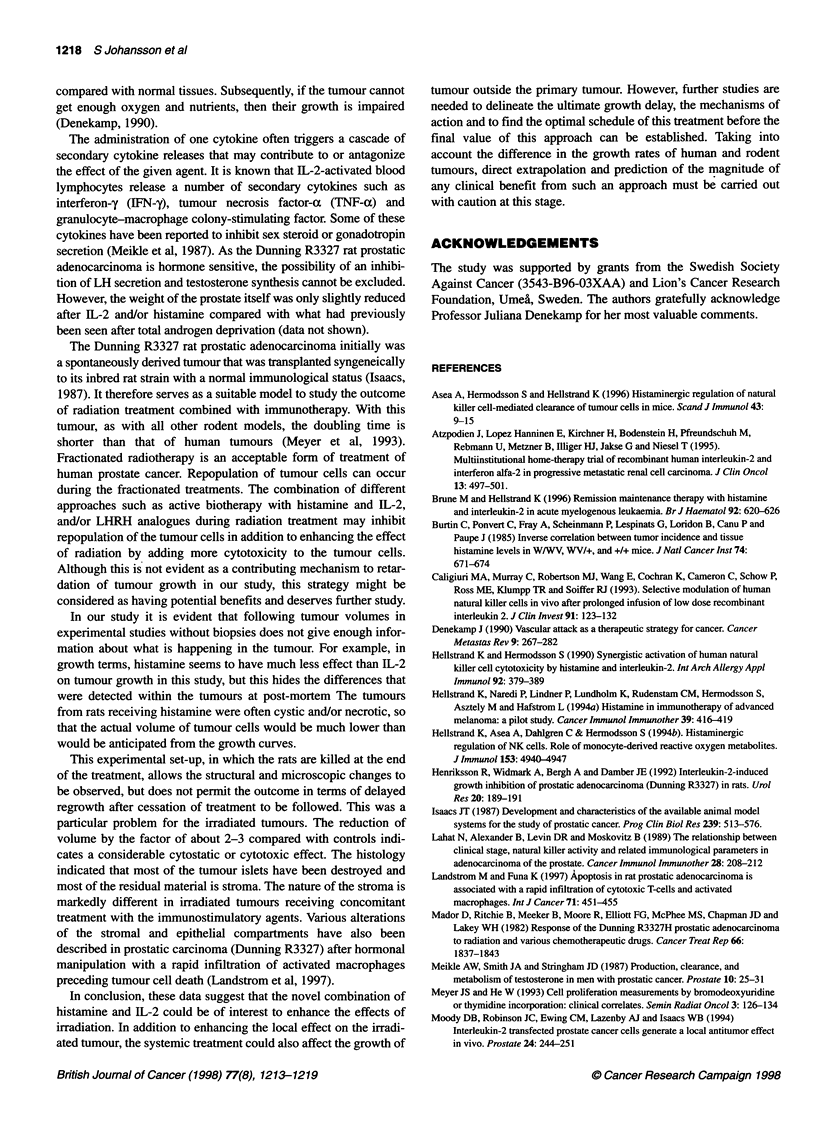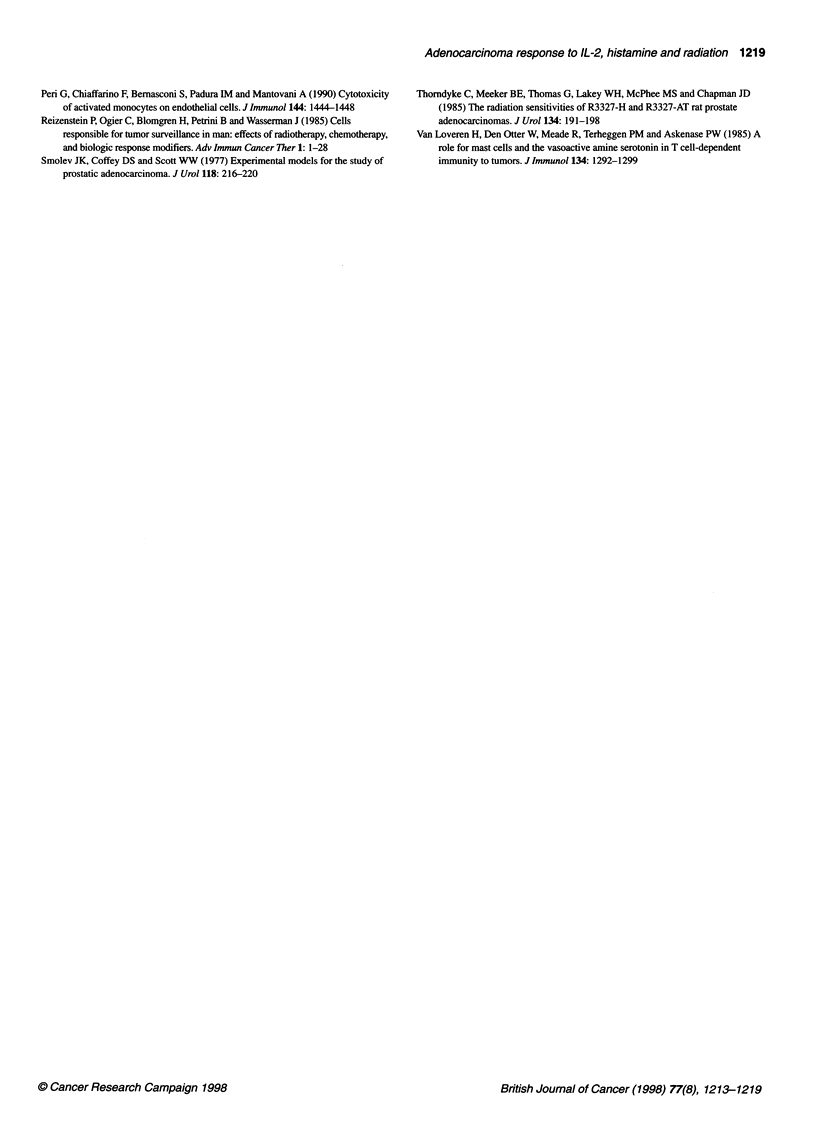# The response of Dunning R3327 prostatic adenocarcinoma to IL-2, histamine and radiation.

**DOI:** 10.1038/bjc.1998.205

**Published:** 1998-04

**Authors:** S. Johansson, M. LandstrÃ¶m, K. Hellstrand, R. Henriksson

**Affiliations:** Department of Oncology, University of UmeÃ¥, Ludwig Institute for Cancer Research, Sweden.

## Abstract

**Images:**


British Journal of Cancer (1998) 77(8), 1213–1219
© 1998 Cancer Research Campaign

### The response of Dunning R3327 prostatic adenocarcinoma to IL-2, histamine and radiation

**S Johansson[1], M Landström[2,3] K Hellstrand[4] and R Henriksson[1]**

Departments of [1]Oncology and [2]Pathology, University of Umeå, Ludwig Institute for Cancer Research, [3]Biomedical Centre, Uppsala; and [4]Department of Virology, University of Gothenburg, Sweden

**Summary** A syngeneic, androgen-sensitive Dunning R3327 rat prostatic adenocarcinoma was transplanted bilaterally in the flanks of male Copenhagen Fisher rats. Approximately 3 months after implantation, when the tumours had a median volume of 150 mm$^3$, one group of rats was treated with histamine alone (4 mg kg$^{-1}$ subcutaneously on week days), another group with human recombinant interleukin 2 (IL-2) alone (425 IU kg$^{-1}$ continuous infusion) and a third group with both histamine and IL-2 during 6 weeks. Tumours on one flank were irradiated (6 Gy once daily for 3 days to a total dose of 18 Gy) beginning 1 week after the onset of treatment with histamine and / or IL-2. The contralateral tumour served as the intra-animal control. The tumour volumes were determined weekly. The growth curves showed that all three drug treatments were effective in delaying growth, but when used individually did not cause tumour shrinkage. Radiation was the most effective single agent, but when used alone the shrinkage did not occur until 2 weeks after irradiation. When combined with the drugs, more rapid and extensive growth delay and/or shrinkage was seen. The growth curves showed clear differences between the different treatments. The combination of the three agents was the most effective of all. The most striking difference between radiation alone and radiation plus biotherapy was the time at which a tumour response was detectable. Thus, active biotherapy alone and especially in a combination with histamine and radiotherapy warrants further investigation as a potential therapeutic approach to prostate cancer.

**Keywords:** histamine; interleukin 2; radiation; Dunning R3327 prostatic carcinoma

The pivotal role of the cytokine interleukin 2 (IL-2) in the immune system has promoted trials of IL-2 in patients with solid cancers such as melanoma and renal cell carcinoma (Atzpodien et al, 1995). IL-2 effectively stimulates the anti-tumour activity of natural killer (NK) cells and other cytotoxic cells in the majority of treated patients (Caligiuri et al, 1993), but the effects on tumour burden and on the survival of patients have, as yet, been disappointing. The detailed mechanisms that explain the poor clinical outcome are not known.

In the inflammatory tissue adjacent to tumours, the predominant leucocytes are usually phagocytes such as monocytes and granulocytes. Recent studies suggest that NK cells are only weakly activated by IL-2 in the presence of phagocytes in vitro, presumably because of the production of an inhibitory signal from the phagocytes (Hellstrand et al, 1994*a*). Histamine, a biogenic amine, counteracts this phagocyte-derived suppressive signal. Thereby, histamine may synergize with IL-2 to induce NK cell-mediated killing of a variety of cultured tumour cells in vitro (Hellstrand and Hermodsson, 1990; Hellstrand et al, 1994*b*). Treatment of mice with histamine also enhances the IL-2-induced activation of NK cells in vivo (Asea et al, 1996) and addition of histamine to conventional IL-2 therapy has shown initial promise in patients with metastatic melanoma (Hellstrand et al, 1994*a*) and acute myelogenous leukaemia (Brune and Hellstrand, 1996).

*Received 30 April 1997*
*Revised 24 June 1997*
*Accepted 3 October 1997*

*Correspondence to:* S Johansson, Department of Oncology, Umeå University Hospital, S-901 85 Umeå, Sweden

It is also of interest to observe that radiation reduces the T-helper cell function, but *not* that of macrophages or NK cells (Reizenstein et al, 1985).

The Dunning R3327 rat prostatic adenocarcinoma arose spontaneously in a male Copenhagen rat and has been carried subcutaneously as several distinct tumour cell lines. It is well differentiated, considered to be a non-immunogenic, low-grade and hormone-sensitive prostate adenocarcinoma and a suitable model for established human prostatic carcinoma (Smolev et al, 1977). The tumours have previously been shown to be radiosensitive (Thorndyke et al, 1985). Previous studies have also shown that IL-2 significantly delays tumour growth in this in vivo model, even when treatment is initiated several months after tumour implantation (Henriksson et al, 1992; Moody et al, 1994).

In the present study, we have investigated whether treatment with histamine alone, IL-2 alone or IL-2 in a combination with histamine influences the growth of tumours receiving drugs alone or concomitant with radiation treatment of Dunning R3327 prostatic adenocarcinoma in rats. The growth pattern during treatment, the volume and the appearance of the tumours have been evaluated both macroscopically and microscopically at sacrifice.

### MATERIALS AND METHODS

### Animals

A 1-mm$^3$ cube of prostatic adenocarcinoma tissue (Dunning R3327) was implanted bilaterally into each flank of 10-week-old, male offspring of Copenhagen X Fisher F1 rats at the Department of Physiology, University of Umeå, Sweden. The tumours were originally obtained from Dr NH Altman (Organ System Program of the National Cancer Institute, USA). The animals were housed in a controlled environment (12 h light/12 h dark) with pellets and water freely available. Approximately 3 months after implantation, when the weight of the rats had reached 460 g (430–480) and the tumours had a median volume of 150 mm$^3$ (122–340), eight rats were chosen at random for IL-2 treatment, eight rats for histamine treatment, eight rats for treatment with a combination of IL-2 and histamine treatment, and six rats were used as controls.

### IL-2 and histamine administration

IL-2 was continuously delivered as a subcutaneous infusion using an Alzet micro-osmotic pump (model 2002) implanted on the neck for 6 weeks of treatment (Alza, Palo Alto, CA, USA). This model makes it possible to deliver a steady concentration of drug (0.5 µl h$^{-1}$ for 14 days). The pumps were filled with IL-2 solution in a concentration of 1.8 × 10$^6$ IU in 200 µl, which delivers a daily dose of 425 IU kg$^{-1}$. Every 2 weeks the pumps were replaced, providing fresh solution.

Histamine was administrated subcutaneously five times a week (Monday to Friday) in a dose of 4 mg kg$^{-1}$, starting 2 days after the implantation of IL-2 pumps and delivered until the termination of the experiment (6 weeks).

### Radiation therapy procedures

The right side of each tumour-bearing rat was irradiated 7 days after the implantation of IL-2 pumps and the contralateral tumours were used as the intra-animal controls. The rats were irradiated with a fractionated schedule. A dose of 6 Gy was given on three consecutive days with X-rays from a medical linear accelerator (6 MV). Focus to skin distance was 100 cm. The rats were firmly anaesthetized using methohexital (Brietal) administrated intraperitoneally and fixed in a plastic net mould during each irradiation period. During irradiation the rats were checked through a TV camera, and if they moved the treatment was temporarily interrupted to realign them. The total radiation field was 5 × 10 cm as two rats were irradiated at the same time, and the rats were also carefully shielded to reduce the scattered dose of radiation to normal tissues outside the treatment field, trying to avoid irradiating the intestine.

### Tumour growth

The tumours reached treatment size approximately 3 months after inoculation. They were measured weekly, always by the same person, with callipers in three mutually orthogonal directions. Tumour volumes were calculated assuming spherical geometry, using the formula $V = \pi/6 \times D_1 \times D_2 \times D_3$. The day of implantation of IL-2 pumps was designated as day 0. Tumour response to various treatments is shown graphically as mean tumour volume (of the six to eight tumours per treatment group) as a function of time after the start of treatment. The weight of the rats was also measured weekly.

### Statistical analyses

Values are expressed as means ± s.e.m unless otherwise indicated. For comparisons between groups the Mann–Whitney *U*-test was used to obtain the *P*-values.

### RESULTS

### General effects

No obvious toxicity was observed during the treatment period; in particular, there were no signs of radiation-induced gastrointestinal complications. The body weight of rats 1 week before and for 5 consecutive weeks after radiation treatment was measured. A 7–10% weight loss was observed 1 week after irradiation in all groups. They all recovered, except the total combined treatment group in which the weight loss persisted throughout the observation period.

### Tumour growth

Figure 1 shows the growth curves of the groups of tumours receiving a single therapy. The untreated Dunning R3327 tumours show continuous growth over the 6-week period, with a volume doubling time of 2 weeks, slowing to more than 4 weeks as the experiment progressed. Radiation is the most effective single agent, causing regression below its initial treatment size but with regrowth commencing at weeks 5 and 6. Histamine alone is the least effective treatment. IL-2 is intermediate.

Figure 2A–D shows the effect of the various combinations. If histamine and IL-2 are combined, the end result is similar to IL-2 alone, although at early times the combination appears more effective. If IL-2 is added to 18 Gy, the net result is similar to 18 Gy alone, even although pretreatment for 1 week with IL-2 made the tumours smaller at the time of irradiation. If histamine is added to 18 Gy, the response is similar up to week 4, but appears more effective at late times. The most effective treatment is the combination of all three agents (histamine, IL-2 and radiation), which is significantly greater than the response to radiation alone at 2 weeks, but not at 6 (Table 1).

Figure 3 displays a comparison of volumes of the eight groups of tumours at week 2 and week 6. Adjacent columns allow comparison of the irradiated and unirradiated tumours on the same rats, exposed to precisely the same drug regimes. The drugs reduce the volume of the tumours compared with those in an untreated group of rats. The addition of the drugs to radiotherapy also deceased the size, although the difference was not statistically significant at later times (see Table 1).

At the end of the experiment, 5 weeks after irradiation, the control tumours had grown to 3.5 times the initial volume. In the irradiated groups none of the tumours had reached double the initial tumour volume. In every animal the irradiated tumours were considerably smaller than the unirradiated contralateral tumours.

Figure 4 shows the calculated time to reach 175%, 200% and 250% of the initial volume. This time was obtained from the growth curves. The volume doubling time is 14 days in the control group, and histamine alone increased the time to double to 23 days, IL-2 alone to 32 days, whereas IL-2 and histamine treatment together increased the doubling time to 37 days. The volume doubling time of the irradiated groups could not be evaluated as none of the irradiated tumours had reached double size when the study was finished and the animals were killed.

### Tumour morphology

Figure 5 shows photomicrographs of control, untreated tumours and those irradiated alone or with single or combination drugs.

© Cancer Research Campaign 1998

**Figure 1** Growth curves of Dunning R3327 tumours exposed to single agents, as indicated. Drugs given continuously from day 0, radiation given as 3 × 6 Gy on days 7–9. △, Control; ▽, histamine; ○, IL-2; ▲, radiation therapy

**Figure 2** Four panels to illustrate the tumour growth curves after single agents and combination of two or three agents during the treatment period. Drugs given continuously from day 0, radiation given as 3 × 6 Gy on days 7–9. (**A**) ▽, Histamine; ○, IL-2; □, histamine + IL-2. (**B**) ○, IL-2; ▲, radiation therapy; ●, IL-2 + radiation therapy. (**C**) ▽, Histamine; ▲, radiation therapy; ▼, histamine + radiation therapy. (**D**) □, Histamine + IL-2; ▲, radiation therapy; ■, histamine + IL-2 + radiation therapy

© Cancer Research Campaign 1998

**Table 1** Statistical analysis of the results obtained in Figures 2 and 3 using the Mann–Whitney *U*-test

| | Assay time | Radiation | Agent used | | |
|---|---|---|---|---|---|
| | | | **IL-2** | **Histamine** | **IL-2 + Histamine** |
| Versus untreated control tumours | 2 weeks | n.s. | $P < 0.037$ | $P < 0.053$ | $P < 0.009$ |
| | 6 weeks | $P < 0.015$ | NS | NS | NS |
| | | | **RT+IL-2** | **RT+Histamine** | **RT+IL–2+Histamine** |
| Versus radiation only | 2 weeks | – | $P < 0.021$ | $P < 0.001$ | $P < 0.002$ |
| | 6 weeks | – | NS | NS | NS |

**Figure 3** Histogram to show the relative volumes of tumours (on opposite flanks of rats) treated with drugs alone or in combination with radiotherapy at (**A**) 2 weeks after initiating drug therapy and at (**B**) 6 weeks, i.e. at termination of experiment. □, Radiation therapy; ■, no radiation therapy

**Figure 4** Delay in regrowth of tumours treated with drugs alone. Time taken to reach different end point volumes are shown in the three sections. □, Control; ■, histamine; ▧, IL-2; ▨, histamine + IL-2

Macroscopically at sacrifice, it was evident that histamine alone and in combinations with the other modalities caused alterations in the tumour tissue with the appearance of haemorrhagic cystic structures. This was most evident in the tumours receiving the full combination. The untreated tumours in rats in which the contralateral tumour was irradiated can be regarded as untreated controls for comparative morphological analysis.

Microscopically, these control tumours were composed of a high density of glandular structure embedded in a small amount of stroma (Figure 5A). The irradiated tumours showed a clear alteration of the morphology with a decreased amount of tumour cells surrounded by a cell-rich stroma (Figure 5B). In irradiated tumours treated with histamine, an even more pronounced reduction in the number of tumour cells was found when compared with control tumours and the contralateral, non-irradiated tumours. The tumour stroma was rich in collagen fibres with far fewer scattered cells (Figure 5C). In irradiated tumours treated with IL-2, a reduction in the number of tumour cells was found and the tumour stroma consisted mainly of fibrous tissue with scattered stromal cells (Figure 4D). The major difference in these irradiated tumours is in the cellularity of the stroma. Irradiated tumours treated with both IL-2 and histamine were most dramatically damaged by the treatment. In some tumours even large areas resembling the picture of coagulative necrosis were observed (Figure 5E).

### DISCUSSION

This study for the first time, as far as we know, demonstrates that IL-2, especially in combination with histamine, alters the time scale of response to radiation. The results are in accordance with earlier observations that a direct and specific manipulation of the

© Cancer Research Campaign 1998

**Figure 5** Tumour histopathology at 5 weeks after irradiation of the tumours treated with histamine and/or IL-2. (**A**) Control, not irradiated; (**B**) irradiated; (**C**) histamine and irradiated; (**D**) IL-2 and irradiated; (**E**) histamine and IL-2 and irradiated immune system can mediate growth delay of different kinds of established tumours in experimental models as well as in humans, including prostatic carcinoma (Mador et al, 1982; Lahat et al, 1989; Henriksson et al, 1992).

The present results showed that the three agents given alone or in various combinations affected both the growth rate of the tumours during the experimental period and the morphology at sacrifice. Each agent was effective as a single agent, in the increasing order, histamine, IL-2 and radiation. When combined, histamine and IL-2 were more effective than histamine and IL-2 alone, but less effective than radiation alone. However, when the combination treatment with histamine and IL-2 was added to radiation, the tumour growth was most retarded. From the histology, the most effective was the treatment with all three agents together.

Previous experimental studies, also in accordance with our data, have shown that in histamine-treated animals large and numerous foci of haemorrhagic necroses were found (Burtin et al, 1985). This was evident macroscopically in our study, with an increased frequency of haemorrhagic cystic structures in all groups that received histamine. The observation might indicate vascular damage due to the histamine administration itself. An increased vascular permeability at the site of tumour growth may facilitate local entry of sensitized T cells that are activated to produce lymphokines. These lymphokines may attract leucocytes from the circulation with a potential anti-tumour activity such as macrophages and NK cells (Van Loveren et al, 1985). Moreover, activated macrophages have been reported to have cytotoxic effects also on endothelial cells (Peri et al, 1990). The fact that cytokines cause changes in blood pressure, vascular permeability and other damage to both small and large blood vessels cannot be ignored either. Decreased blood pressure may result in a decreased blood flow in tumours (steal effect), and an increased vascular permeability caused by both histamine and IL-2 could give rise to an increased interstitial pressure. These factors could be involved in decreasing and/or shutting off the blood flow in tumours compared with normal tissues. Subsequently, if the tumour cannot get enough oxygen and nutrients, then their growth is impaired (Denekamp, 1990).

The administration of one cytokine often triggers a cascade of secondary cytokine releases that may contribute to or antagonize the effect of the given agent. It is known that IL-2-activated blood lymphocytes release a number of secondary cytokines such as interferon-γ (IFN-γ), tumour necrosis factor-α (TNF-α) and granulocyte–macrophage colony-stimulating factor. Some of these cytokines have been reported to inhibit sex steroid or gonadotropin secretion (Meikle et al, 1987). As the Dunning R3327 rat prostatic adenocarcinoma is hormone sensitive, the possibility of an inhibition of LH secretion and testosterone synthesis cannot be excluded. However, the weight of the prostate itself was only slightly reduced after IL-2 and/or histamine compared with what had previously been seen after total androgen deprivation (data not shown).

The Dunning R3327 rat prostatic adenocarcinoma initially was a spontaneously derived tumour that was transplanted syngeneically to its inbred rat strain with a normal immunological status (Isaacs, 1987). It therefore serves as a suitable model to study the outcome of radiation treatment combined with immunotherapy. With this tumour, as with all other rodent models, the doubling time is shorter than that of human tumours (Meyer et al, 1993). Fractionated radiotherapy is an acceptable form of treatment of human prostate cancer. Repopulation of tumour cells can occur during the fractionated treatments. The combination of different approaches such as active biotherapy with histamine and IL-2, and/or LHRH analogues during radiation treatment may inhibit repopulation of the tumour cells in addition to enhancing the effect of radiation by adding more cytotoxicity to the tumour cells. Although this is not evident as a contributing mechanism to retardation of tumour growth in our study, this strategy might be considered as having potential benefits and deserves further study.

In our study it is evident that following tumour volumes in experimental studies without biopsies does not give enough information about what is happening in the tumour. For example, in growth terms, histamine seems to have much less effect than IL-2 on tumour growth in this study, but this hides the differences that were detected within the tumours at post-mortem The tumours from rats receiving histamine were often cystic and/or necrotic, so that the actual volume of tumour cells would be much lower than would be anticipated from the growth curves.

This experimental set-up, in which the rats are killed at the end of the treatment, allows the structural and microscopic changes to be observed, but does not permit the outcome in terms of delayed regrowth after cessation of treatment to be followed. This was a particular problem for the irradiated tumours. The reduction of volume by the factor of about 2–3 compared with controls indicates a considerable cytostatic or cytotoxic effect. The histology indicated that most of the tumour islets have been destroyed and most of the residual material is stroma. The nature of the stroma is markedly different in irradiated tumours receiving concomitant treatment with the immunostimulatory agents. Various alterations of the stromal and epithelial compartments have also been described in prostatic carcinoma (Dunning R3327) after hormonal manipulation with a rapid infiltration of activated macrophages preceding tumour cell death (Landstrom et al, 1997).

In conclusion, these data suggest that the novel combination of histamine and IL-2 could be of interest to enhance the effects of irradiation. In addition to enhancing the local effect on the irradiated tumour, the systemic treatment could also affect the growth of tumour outside the primary tumour. However, further studies are needed to delineate the ultimate growth delay, the mechanisms of action and to find the optimal schedule of this treatment before the final value of this approach can be established. Taking into account the difference in the growth rates of human and rodent tumours, direct extrapolation and prediction of the magnitude of any clinical benefit from such an approach must be carried out with caution at this stage.

### ACKNOWLEDGEMENTS

The study was supported by grants from the Swedish Society Against Cancer (3543-B96-03XAA) and Lion's Cancer Research Foundation, Umeå, Sweden. The authors gratefully acknowledge Professor Juliana Denekamp for her most valuable comments.

### REFERENCES

Asea A, Hermodsson S and Hellstrand K (1996) Histaminergic regulation of natural killer cell-mediated clearance of tumour cells in mice. *Scand J Immunol* **43**: 9–15

Atzpodien J, Lopez Hanninen E, Kirchner H, Bodenstein H, Pfreundschuh M, Rebmann U, Metzner B, Illiger HJ, Jakse G and Niesel T (1995). Multiinstitutional home-therapy trial of recombinant human interleukin-2 and interferon alfa-2 in progressive metastatic renal cell carcinoma. *J Clin Oncol* **13**: 497–501.

Brune M and Hellstrand K (1996) Remission maintenance therapy with histamine and interleukin-2 in acute myelogenous leukaemia. *Br J Haematol* **92**: 620–626

Burtin C, Ponvert C, Fray A, Scheinmann P, Lespinats G, Loridon B, Canu P and Paupe J (1985) Inverse correlation between tumor incidence and tissue histamine levels in W/WV, WV/+, and +/+ mice. *J Natl Cancer Inst* **74**: 671–674

Caligiuri MA, Murray C, Robertson MJ, Wang E, Cochran K, Cameron C, Schow P, Ross ME, Klumpp TR and Soiffer RJ (1993). Selective modulation of human natural killer cells in vivo after prolonged infusion of low dose recombinant interleukin 2. *J Clin Invest* **91**: 123–132

Denekamp J (1990) Vascular attack as a therapeutic strategy for cancer. *Cancer Metastas Rev* **9**: 267–282

Hellstrand K and Hermodsson S (1990) Synergistic activation of human natural killer cell cytotoxicity by histamine and interleukin-2. *Int Arch Allergy Appl Immunol* **92**: 379–389

Hellstrand K, Naredi P, Lindner P, Lundholm K, Rudenstam CM, Hermodsson S, Asztely M and Hafstrom L (1994*a*) Histamine in immunotherapy of advanced melanoma: a pilot study. *Cancer Immunol Immunother* **39**: 416–419

Hellstrand K, Asea A, Dahlgren C & Hermodsson S (1994*b*). Histaminergic regulation of NK cells. Role of monocyte-derived reactive oxygen metabolites. *J Immunol* **153**: 4940–4947

Henriksson R, Widmark A, Bergh A and Damber JE (1992) Interleukin-2-induced growth inhibition of prostatic adenocarcinoma (Dunning R3327) in rats. *Urol Res* **20**: 189–191

Isaacs JT (1987) Development and characteristics of the available animal model systems for the study of prostatic cancer. *Prog Clin Biol Res* **239**: 513–576.

Lahat N, Alexander B, Levin DR and Moskovitz B (1989) The relationship between clinical stage, natural killer activity and related immunological parameters in adenocarcinoma of the prostate. *Cancer Immunol Immunother* **28**: 208–212

Landstrom M and Funa K (1997) Apoptosis in rat prostatic adenocarcinoma is associated with a rapid infiltration of cytotoxic T-cells and activated macrophages. *Int J Cancer* **71**: 451–455

Mador D, Ritchie B, Meeker B, Moore R, Elliott FG, McPhee MS, Chapman JD and Lakey WH (1982) Response of the Dunning R3327H prostatic adenocarcinoma to radiation and various chemotherapeutic drugs. *Cancer Treat Rep* **66**: 1837–1843

Meikle AW, Smith JA and Stringham JD (1987) Production, clearance, and metabolism of testosterone in men with prostatic cancer. *Prostate* **10**: 25–31

Meyer JS and He W (1993) Cell proliferation measurements by bromodeoxyuridine or thymidine incorporation: clinical correlates. *Semin Radiat Oncol* **3**: 126–134

Moody DB, Robinson JC, Ewing CM, Lazenby AJ and Isaacs WB (1994) Interleukin-2 transfected prostate cancer cells generate a local antitumor effect in vivo. *Prostate* **24**: 244–251

 © Cancer Research Campaign 1998

Peri G, Chiaffarino F, Bernasconi S, Padura IM and Mantovani A (1990) Cytotoxicity of activated monocytes on endothelial cells. *J Immunol* **144**: 1444–1448
Reizenstein P, Ogier C, Blomgren H, Petrini B and Wasserman J (1985) Cells responsible for tumor surveillance in man: effects of radiotherapy, chemotherapy, and biologic response modifiers. *Adv Immun Cancer Ther* **1**: 1–28
Smolev JK, Coffey DS and Scott WW (1977) Experimental models for the study of prostatic adenocarcinoma. *J Urol* **118**: 216–220
Thorndyke C, Meeker BE, Thomas G, Lakey WH, McPhee MS and Chapman JD (1985) The radiation sensitivities of R3327-H and R3327-AT rat prostate adenocarcinomas. *J Urol* **134**: 191–198
Van Loveren H, Den Otter W, Meade R, Terheggen PM and Askenase PW (1985) A role for mast cells and the vasoactive amine serotonin in T cell-dependent immunity to tumors. *J Immunol* **134**: 1292–1299

© Cancer Research Campaign 1998